# LADRC-based grid-connected control strategy for single-phase LCL-type inverters

**DOI:** 10.1371/journal.pone.0303591

**Published:** 2024-05-15

**Authors:** Zhi Feng, Xiaojie Zhou, Chan Wang, Weizhou Huang, Ziqi Chen

**Affiliations:** Department of Electrical and Electronic Engineering, Anhui Science and Technology University, Bengbu, China; Vellore Institute of Technology, INDIA

## Abstract

To ensure that grid-connected currents are of high quality, it is crucial to optimize the dynamic performance of grid-connected inverters and their control. This study suggests using a combination of reduced-order linear active disturbance rejection control (LADRC) and a Proportional-Integral (PI) controller. By applying this control strategy to a single-phase photovoltaic grid-connected system, the system’s ability to suppress grid harmonics is significantly improved. The validity and effectiveness of this control approach have been confirmed through simulations and experiments. The results show that the LADRC-based control system is robust and capable of rejecting disturbances, resulting in a significant reduction in the Total Harmonic Distortion (THD) of grid-connected currents. Comparative analysis with traditional control methods demonstrates the superior performance of the proposed approach.

## Introduction

As the world enters a new industrial era led by clean energy technologies, the gradual shift from traditional fossil fuels to renewable energy has become an unavoidable trend [1]. Solar energy, as a type of renewable energy, has garnered significant attention from scholars around the globe [2]. Grid-connected inverters are a key part of photovoltaic power generation systems. They play a crucial role in directly connecting solar power generation units to the grid, and their performance directly affects the quality of the final current [3–5].

Different control strategies and filter topologies have become the focus of research to achieve high-quality output current from the inverter. Currently, some commonly employed control strategies for inverters are PI control, PR control, and repetitive control [6–8]. PR control has emerged as the most promising current regulator type for single-phase inverters due to its ability to introduce infinite gain at a selected resonance frequency, such as the fundamental frequency. This unique characteristic allows PR control to eliminate steady-state errors that cannot be achieved by conventional PI control [9]. However, single repetitive control exhibits poor dynamic performance [10]. Due to its high steady-state accuracy and excellent dynamic performance, many inverter systems use the PI+repetitive control approach [11, 12]. During dynamic processes, control coupling between the PI and repetitive controllers can distort the grid-connected current. The mechanism of control coupling was analyzed, and a grid-connected inverter control method based on an enhanced version of repetitive control was proposed [13]. Reference [14] proposes an enhanced feedforward controller for microgrid systems to improve the grid synchronization ability, enhance its power injection/absorption capabilities, and maintain stability under weak grid conditions. Reference [15] proposes a Hilbert transform weight factor (HTWF) based control strategy for improving the reliability of grid-integrated solar photovoltaic (PV) systems. To eliminate the penetration problem of traditional voltage inverters, the staggered inverter topology is proposed in reference [16] to improve system efficiency. Reference [17] presents a new multilevel inverter and a strong control system to improve the fault ride-through and power quality capabilities of DFIG-based wind turbines. Recent advances in lithium-battery and super-capacitor technologies have allowed for their use as a hybrid energy storage system for the DC-power supply of dynamic voltage restorer, with the proposed fractional-order super-twisting sliding mode control effectively compensating for system uncertainties and nonlinearities [18]. Reference [19] introduces a novel approach using a Half-Cascaded Multilevel Inverter (HCMLI) coupled to a photovoltaic power source as an AC-voltage synthesizer for a Dynamic Voltage Restorer (DVR), aiming to enhance the compensation of voltage disturbances in a cost-effective and eco-friendly manner. Reference [20] introduces a novel multi-level inverter (OCAMLI) to enhance the performance of the Dynamic Voltage Restorer (DVR) in compensating voltage sags, swells, and harmonics, compared to conventional DVR. Professor Han Jingqing from China proposed ADRC as a modern control method aiming to refine PID controllers’ essence [21]. In engineering control, ADRC has been widely applied in various domains and has gained attention from many scholars both domestically and internationally. However, its application in grid-connected inverters still needs to be improved, requiring further research and practice to promote and refine its usage in this field. Reference [22] addresses the issues of insufficient disturbance rejection performance and control accuracy in the traditional dual-loop PI control strategy for grid-connected inverters. An enhanced ADRC technique utilizing a Nonlinear Extended State Observer (NLESO) has been proposed to optimize DC bus voltage control performance. However, due to the uncertainty of the system model and the complexity of external disturbances, this controller may exhibit instability or poor performance in practical applications. Reference [23] provides a detailed introduction to the parameter tuning of the active disturbance rejection system. In reference [24], a state-space model is developed in the d-q synchronous rotating coordinate system to describe a three-phase *LCL*-type grid-connected inverter. Using the "relative order" of the controlled object, a second-order LADRC is designed. Reference [25] introduces a novel control strategy for photovoltaic inverters; the voltage outer loop is regulated by an enhanced LADRC. The enhanced LADRC strategy comprises conventional PD control algorithms, Linear Extended State Observer (LESO), and a correction module. Compared to traditional LADRC, the improved LADRC exhibits significantly enhanced high-frequency attenuation capabilities. Although enhanced LADRC has significant advantages over high-frequency attenuation, its complexity, and computational requirements can also increase, potentially decreasing real-time performance.

The primary focus of this paper is the design and evaluation of a control strategy for an *LCL* single-phase grid-connected inverter. Specifically, we present a detailed description of the reduced order system model, the design process for LESO, and the control rate LSEF. Furthermore, we compare the performance of the proposed PI-LADRC control strategy with the traditional PI-PR control through comprehensive simulation and experimental results. The verification and validation of the designed control strategy demonstrate its effectiveness in enhancing the performance of the inverter system.

## Materials and methods

This paper describes a model for a single-phase photovoltaic grid-connected inverter. The mathematical representation of the inverter is established and simplified using a reduced-order approach. Moreover, a controller based on a first-order LADRC is designed to regulate the inverter.

### Modeling of single-phase grid-connected inverter

**Fig 1:**
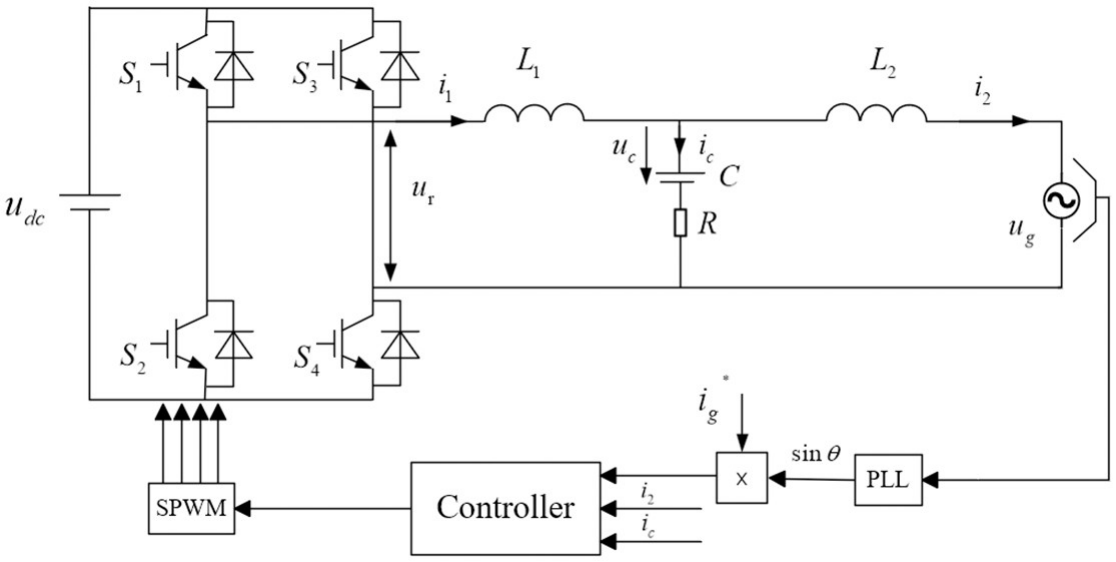
Single-phase *LCL*-type grid-connected inverter.

As depicted in Fig 1, the primary components of the single-phase photovoltaic grid-connected inverter model include a DC-AC inverter and an *LCL* filter. The DC-AC inverter converts the direct current voltage collected by the solar panel into the required grid-connected alternating current voltage. At the same time, the *LCL* filter is primarily responsible for filtering the inverter output voltage and attenuating high-frequency harmonics to ensure the quality of the output voltage and prevent high-frequency interference from affecting the power grid. It mainly comprises an H4 bridge constructed by four IGBT switches Q_1–4_ and an *LCL* filter constructed by inductors *L*_1_, *L*_2_, and capacitor *C*. The currents *i*_1_, *i*_c,_ and *i*_2_ represent the current flowing out of the inverter, The current passing through the capacitor, and the grid-connected current, respectively. The voltages *u*_dc_, *u*_c_, and *u*_g_ represent the DC voltage, the voltage across the capacitor, and the grid-connected voltage, respectively.

In this structure, the inherent passive damping properties of the *LCL* filter help reduce harmonic resonance and improve system stability, thereby lowering the repetition rate. By selecting appropriate inductance and capacitance values, *LCL* filters can naturally dissipate energy and suppress oscillations, effectively enhancing the system’s performance and reliability. This passive approach offers a cost-effective solution for grid-connected inverters.

By conducting an analysis, we can derive the transfer function that relates the grid-connected inverter output voltage *u*_r_ to the grid-connected current *i*_2_ as Eq (1) [21].


$$G_{LCL}(S)=\frac{i_{2}}{u_{r}}=\frac{sRC+1}{s^{3}L_{1}L_{2}C+s^{2}(L_{1}+L_{2})RC+s(L_{1}+L_{2})}$$ (1)

The self-disturbance rejection controller, based on Eq (1), is a 3rd-order controller that involves differentiating multiple state variables. As a result, its structure is relatively complex, and tuning the parameters can take time and effort. Furthermore, high-order self-disturbance rejection controllers require accurate control object models for optimal control performance. To address this issue, a common approach is to use low-order self-disturbance rejection controllers. These controllers treat modeling errors as internal disturbances, eliminating the need for precise modeling of the control object. This approach simplifies the controller structure and minimizes the required tuning parameters, making it more practical for engineering applications. In practical applications, it is common to approximate the third-order system model with a first-order model when designing low-order self-disturbance rejection controllers. This approach helps achieve a balance between control performance and computational complexity. To reduce the order of Eq (1), the Padé approximation method can be utilized, resulting in a transfer function from grid current *i*_2_ to output voltage *u*_r_, as illustrated in Eq (2) [22]. With this transfer function, a first-order self-disturbance rejection controller can be designed.


$$G_{LCL}(S)=\frac{i_{2}}{u_{r}}=\frac{sRC+1}{s^{3}L_{1}L_{2}C+s^{2}(L_{1}+L_{2})RC+s(L_{1}+L_{2})}\approx \frac{1}{s(L_{1}+L_{2})}=G_{Pade-LCL}(S)$$ (2)

**Fig 2:**
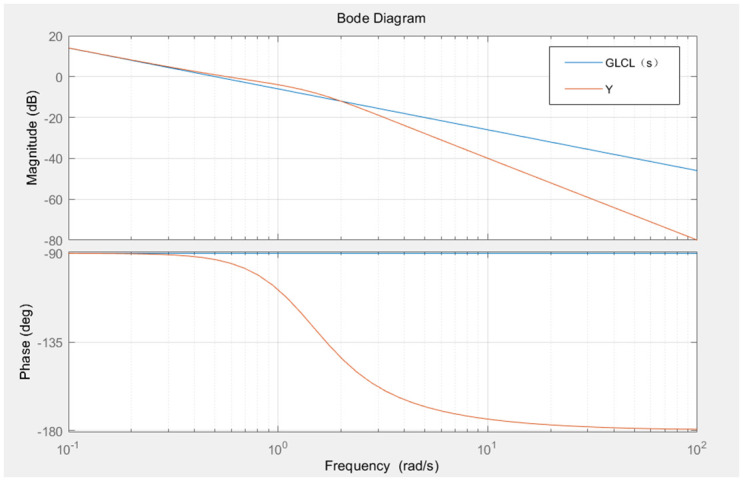
Bode plot of the transfer function.

The Bode plots of the transfer functions for *G*_*LCL*_(*S*) and *G*_*pade*−*LCL*_(*S*) are shown in Fig 2.

From the plot, it is evident that both transfer functions exhibit similar characteristics at low frequencies. Therefore, the main objective of the feedback controller is to correct the low-frequency characteristics of the system. Additionally, from the perspective of considering modeling errors as "generalized disturbances" in the self-disturbance rejection framework, even if there are errors in the reduced-order model at high frequencies, the self-disturbance rejection controller employs a state observer to estimate these errors, which are then compensated for in the compensation stage. This compensation effectively mitigates any negative impact on the system caused by the errors.

The 3rd-order *LCL* grid-connected inverter model can be effectively simplified using order reduction. By employing an extended observer for accurate disturbance estimation and compensation, the first-order self-disturbance rejection control scheme demonstrates comparable performance to the 3rd-order counterpart. This approach offers benefits such as reduced parameter tuning time and improved system controllability in practical engineering applications. The advanced first-order self-disturbance rejection control design focuses on robust disturbance rejection and efficient parameter tuning, providing a promising solution for enhancing system performance and reliability in grid-connected inverters.

### First-order active disturbance rejection control design

To streamline the control system structure of the *LCL* grid-connected inverter, a reduced-order LADRC approach is implemented. This control scheme comprises a LESO and a control law, which integrates a LSEF and an interference compensation term. By employing this control strategy, the system effectively suppresses disturbances and enables accurate state observation.

Eq (2) represents the transfer function of the *LCL*-type grid-connected inverter after the order reduction process. By utilizing this transfer function, it is possible to derive the differential equation governing the behavior of the grid current [23].


$$\frac{di_{2}(t)}{dt}=\frac{1}{L_{1}+L_{2}}u_{r}(t)$$ (3)

Let d(t) denote the unidentified external disturbance in the *LCL* grid-connected system. The inverter’s system model can be redefined as Eq (4).


$${x_{1}}^{\prime }(t)=bu(t)+f_{0}x_{1}(t)+d(t)$$ (4)

In this equation: *x*_1_(*t*) = *i*_2_(*t*) represents the control current, *u*(*t*) denotes the control input variable in the equation. Let *b* = (*b*_0_ + Δ*b*), where *b*_0_ = 1/(*L*_1_ + *L*_2_) represents the known part in the model, and Δ*bu*(*t*) + *f*_0_*i*_2_(*t*) represents the unknown part modeled in the grid-connected inverter, such as parameter deviations caused by perturbations in the filtering parameters and nonlinear characteristics of some power electronic components, etc [24]. Therefore, Eq (4) can be expressed as.


$$\begin{array}{cc} {x_{1}}^{\prime }(t) & =bu(t)+f_{0}x_{1}(t)+d(t)=(b_{0}+\Delta b)u(t)+f_{0}i_{2}(t)+d(t)\\ & =\frac{1}{L_{1}+L_{2}}u(t)+\Delta bu(t)+f_{0}i_{2}(t)+d(t) \end{array}$$ (5)

Based on Eq (5), it can be inferred that the total disturbance present is composed of two parts, namely the external unknown disturbance and the unknown component modeled inside the grid-connected inverter. Eq (6) can be utilized to represent the overall disturbance present within the grid-connected system.


$$f(t)=\Delta bu(t)+f_{0}i_{2}(t)+d(t)$$ (6)

Let the matrix expression of the state vector of the system be *x*_*pade*−*LCL*_(*t*) = [*i*_2_(*t*) *f*(*t*)]^T^, *x*_2_(*t*) = *f*(*t*), where *y*(*t*) is the system’s output, and *x*_2_(*t*) is the augmented state variable. Based on Eqs (5) and (6), The state equation of the grid-connected inverter with reduced order can be represented as follows.


$$\left\{\begin{array}{c} x_{1}(t)=i_{2}(t)\\ {x_{1}}^{\prime }(t)=\frac{1}{L_{1}+L_{2}}u(t)+f(t)\\ f^{\prime }(t)=\frac{df(t)}{dt} \end{array}\right.$$ (7)

Let:

$$A=\left[\begin{array}{cc} 0 & 1\\ 0 & 0 \end{array}\right],B={\left[\frac{1}{L_{1}+L_{2}}0\right]}^{T},C=\left[\begin{array}{cc} 1 & 0 \end{array}\right],E\left[\begin{array}{c} 1\\ 0 \end{array}\right]$$

The analysis results in the state-space formulation of the system with reduced order, which considers the complete disturbance of the system, as demonstrated in Eq (8).


$$\left\{\begin{array}{c} {x_{Pade-LCL}}^{\prime }(t)=Ax_{Pade-LCL}(t)+Bu(t)+Ef^{\prime }(t)\\ y(t)=Cx_{Pade-LCL}(t) \end{array}\right.$$ (8)

#### Design of Linear Extended State Observer (ESO)

The Linear ESO, also known as the Luenberger Observer, is a method used to estimate the state of a system. It can approximate the values of the system’s state vector, even if the system itself does not directly provide measurements of these state variables. The Linear ESO is commonly applied in control systems to achieve state estimation and feedback control.

In the ADRC, *z*_1,_ and *z*_2_ are used to represent the estimated values of the state variables *x*_1_ and *x*_2_, respectively, which are the estimated values of the grid current *i*_2_ and the total disturbance *f*(0) *β* = [*β*_1_ *β*_2_]^T^ is the bandwidth parameter of the LESO. By appropriately choosing the parameters, the observer state variables can be efficiently synchronized with the system’s state variables. Through the utilization of Eq (8) in conjunction with the Linear ESO framework incorporating output correction, we can derive the following expression:

$$z^{\prime }(t)=(A-\beta C)z(t)+Bu(t)+\beta x_{1}(t)$$ (9)

In the context of the LESO, the matrix (A-*β*C) plays a pivotal role in determining the error decay rate of the observer. The eigenvalues of matrix (A-*β*C) directly influence how quickly errors in the state estimation decay over time, which is crucial for the observer’s ability to accurately track the system’s states and mitigate disturbances effectively.

#### Design of Control Law (LSEF)

The LSEF is a design principle or method to develop and improve control systems. LSEF stands for "Lowest State of Energy Flow," which refers to the state of minimum energy flow. In a control system, energy flow refers to input signals passing through various components to the output signal. The key objective of LSEF design is to minimize energy loss and waste, thereby enhancing the efficiency and performance of the control system. This goal is accomplished by optimizing the pathway for signal transmission and minimizing unnecessary energy conversions.

The LSEF control loop for the reduced-order *LCL* three-level grid-connected inverter is represented by Eq (10).

$${\text{u}}_{0}(t)=k_{p}\left[{i_{2}}^{*}-z_{1}(t)\right]$$ (10)

Where: kp is the parameter in the LSEF, and u_0_(t) is the output signal of the LSEF loop. Thus, the control law for the first-order ADRC is given by.


$$\text{u}(t)=\frac{u_{0}(t)-z_{2}(t)}{b_{0}}$$ (11)

**Fig 3:**
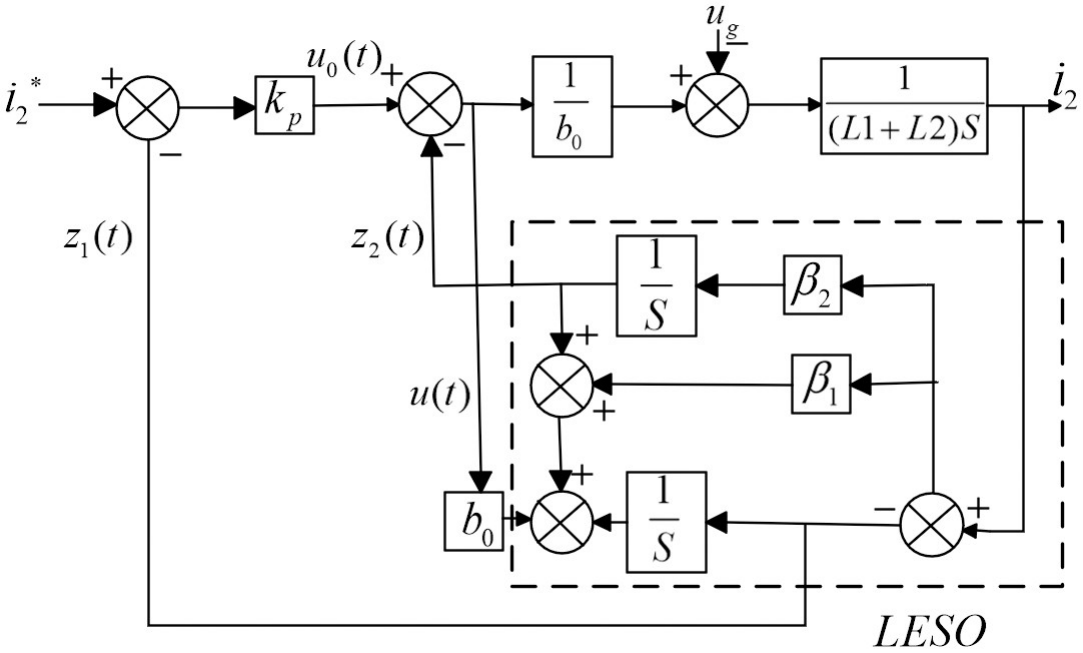
Block diagram of first-order active disturbance rejection control.

Hence, Fig 3 illustrates the block diagram of the first-order ADRC control system specifically designed for the reduced-order *LCL* grid-connected inverter. To simplify the parameter setting process of the LADRC controller, the bandwidth method [23] is commonly used to normalize the controller parameters *k*_p_, *β*. Assuming that *ω*_0_ and *ω*_c_ are the bandwidths of the observer and the controller, then *β* = 2*ω*_0_, and *k*_p_ = *ω*_c_.

The bandwidth approach is frequently employed to streamline the parameter adjustment procedure of the LADRC controller. The LADRC parameters, including the controller and observer bandwidth, can be independently adjusted. Utilizing the system model (illustrated in Fig 3) as a basis, the control gain *b*_o_ has already been determined.

Firstly, an initial value is chosen for *ω*_0_ while keeping *ω*_c_ constant. *ω*_0_ is gradually increased until the noise starts to have a greater impact on the system than what is required. Then, *ω*_c_ is gradually increased while lowering *ω*_0_ if the noise causes fluctuations in the system output. This iterative process continues, adjusting *ω*_0_ and *ω*_c_ accordingly, until the control requirements are met. To achieve precise observation of the complete disturbance by the LESO and enable real-time compensation for the said disturbance, it is common practice to set the observer bandwidth *ω*_0_ within the range of (2–10)*ω*_c_.

#### Design of active disturbance rejection controller of PI-LADRC

**Fig 4:**
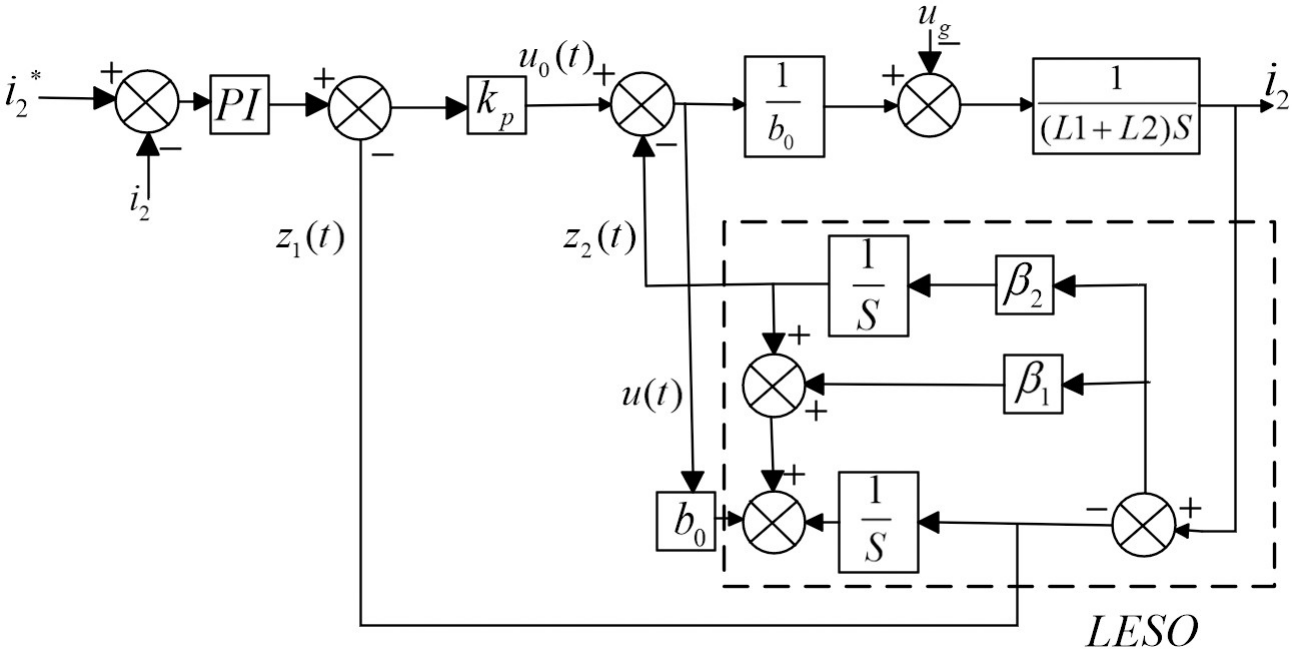
PI-LADRC control diagram.

In PI-LADRC, the proportional-integral controller provides feedback control to stabilize the system and eliminate static errors. The disturbance rejection controller is used to counteract the effects of external disturbances and model uncertainties, thereby improving the robustness and performance of the system. The actual control diagram is shown in Fig 4.

## Results and discussions

To validate the efficacy of the proposed method in controlling grid-connected inverters, a simulation model was constructed using Simulink/MATLAB, as depicted in Fig 4, and a simulation model using PI-PR control was also set up for comparison with PI-LADRC control and traditional PI-PR control in terms of disturbance rejection capability.

**Fig 5:**
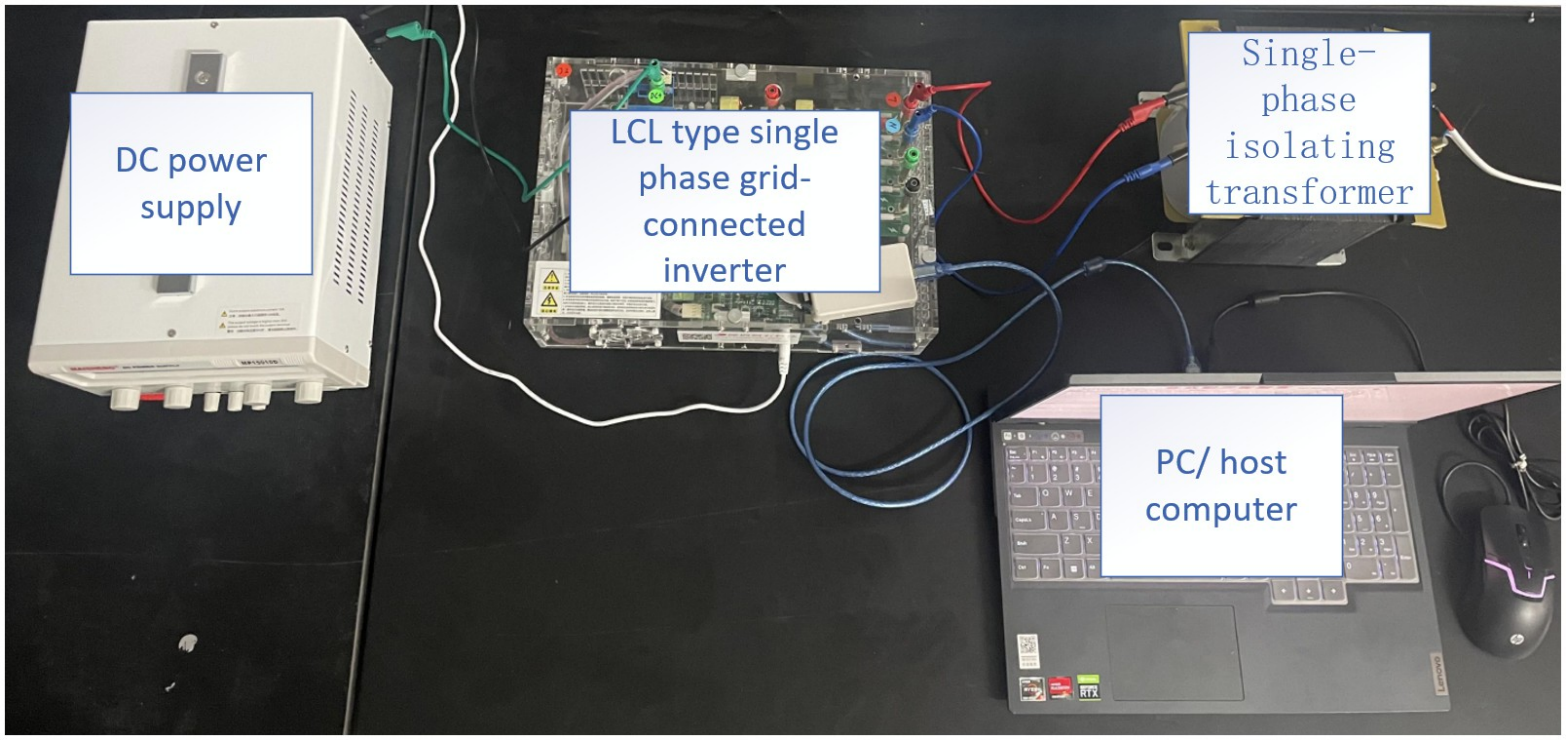
Experimental platform.

In this paper, a 1KW experimental prototype, as shown in Fig 5, was built to validate the actual performance of the PI-LADRC control strategy through experiments. In the experimental prototype, the main control board controller adopted Texas Instruments’ TMS320F28069 chip, the switching tube adopted Infineon’s SiC-MOSFET, and an isolated RS232 communication was used, with commands sent from the upper computer. In the experiment, the parameters are obtained as follows: *k*_p_ = 2000, *ω*_o_ = 4000, *ω*_c_ = 400. The parameters for the PI controller are p = 15, i = 0.7. The experimental prototype parameters are shown in Table 1.

**Table 1:** Main hardware parameters of the experimental platform.

Parameter	Values
DC Side Voltage/V	350
Grid-connected voltage/V	220
Inverter side inductance/mH	2.5
Grid-side inductance/mH	0.34
Filter capacitance/uF	10.5
Switching frequency/kHz	10
Output frequency/Hz	50

Through simulation of the actual operation of PI-LADRC control and traditional PI-PR control, it was found that the error values are shown in Fig 5.

**Fig 6:**
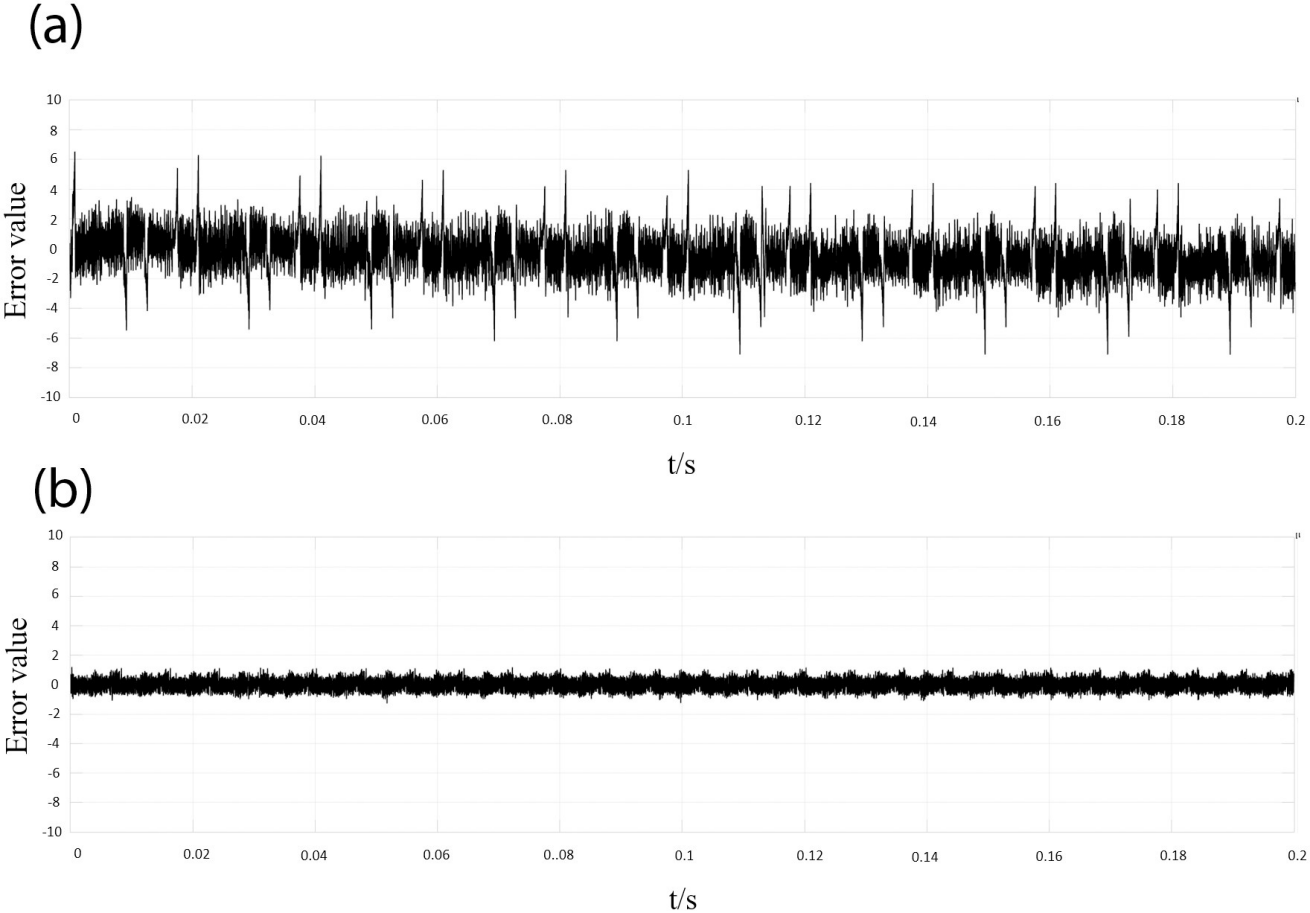
Comparison of error between the two control methods. (a) PI-PR. (b) PI-LADRC.

From Fig 6, it can be observed that the classical PI-PR control has an error of approximately ±6 in terms of disturbance suppression. On the other hand, the proposed PI-LADRC control in this paper exhibits better disturbance rejection capability and control accuracy, with an error within ±1.3.

**Fig 7:**
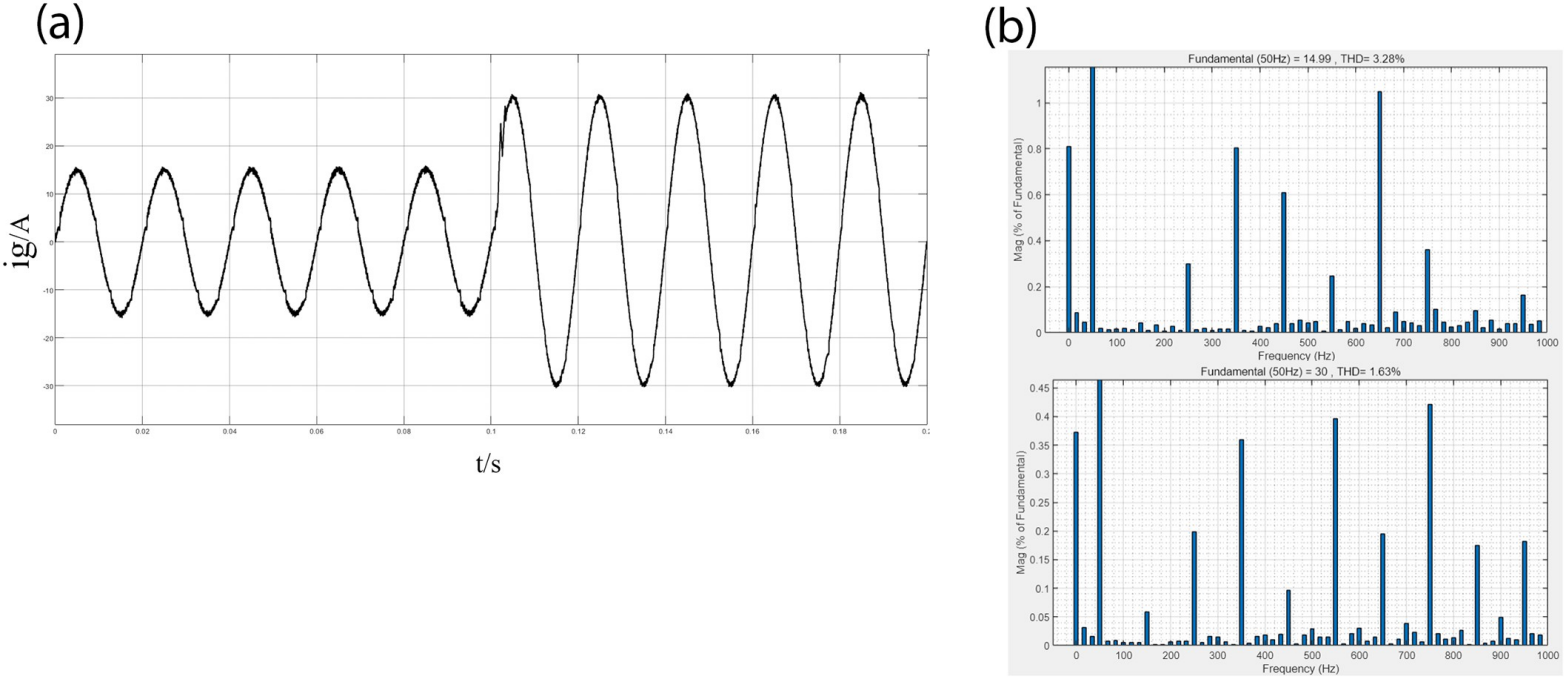
shows the PI-PR grid current and THD analysis before and after the power step change. (a) PI-PR grid current. (b) THD value of grid current before and after the power step change.

**Fig 8:**
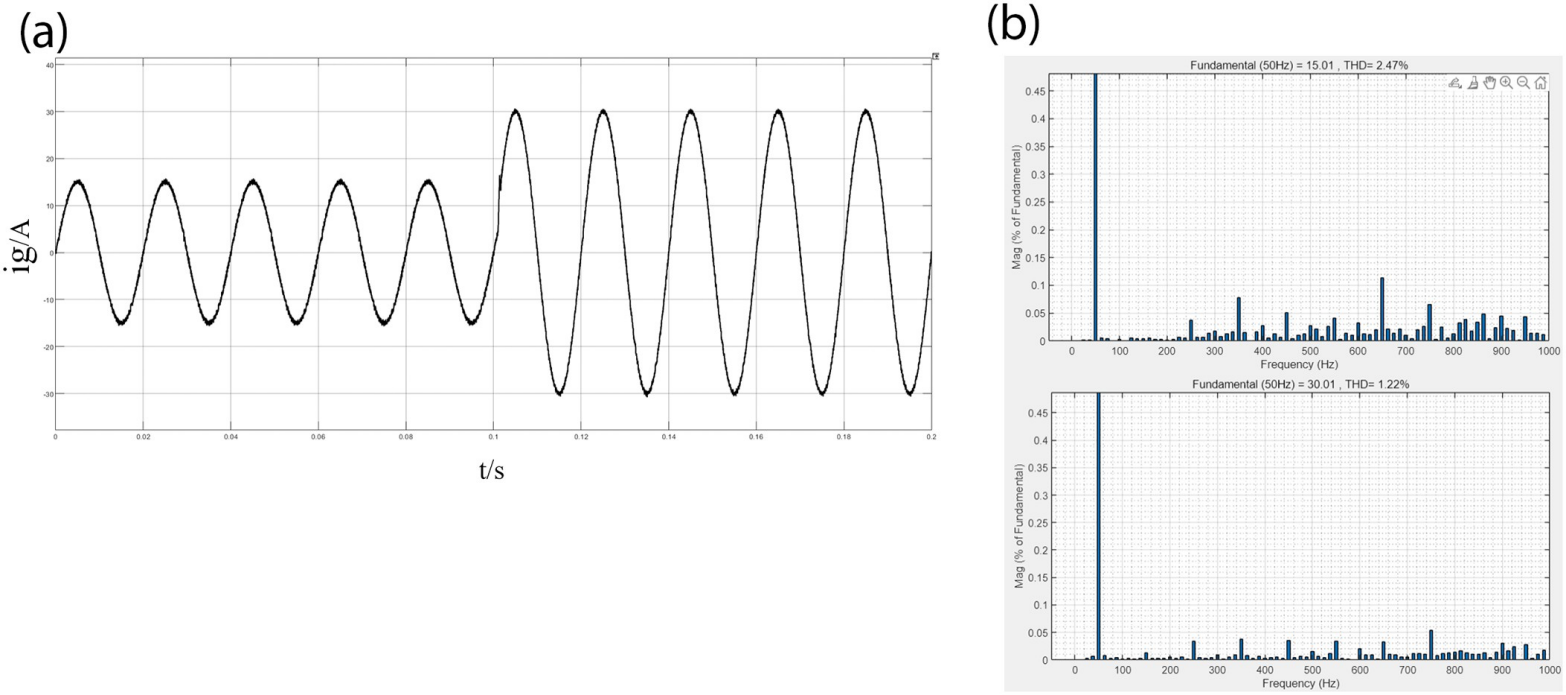
shows the PI-PR grid current and THD analysis before and after the power step change. (a) PI-LADRC grid current. (b) THD value of grid current before and after the power step change.

Figs 7 and 8 show the grid current waveforms and THD analysis of the two control methods when the current reference value ig* changes from 15A to 30A. Comparing Figs 7(a) and 8(a), it can be seen that under PI-PR control, During the power step change, the grid-connected inverter’s output current exhibits oscillations for a specific duration, with a system adjustment time of around 4.7ms. In addition, there is disturbance in the grid current waveform, indicating poor quality of the grid current and insufficient anti-interference capability. However, there is no current overshoot under PI-LADRC control, although there is also short-term oscillation. According to measurements, the system adjustment time is approximately 1.2ms, indicating that the overall dynamic adjustment characteristics of the system are good, with high quality of the grid current and robust anti-interference capability. Furthermore, Through Table 2 and comparing Figs 7(b) and 8(b), it can be seen that the grid-connected system under PI-LADRC control maintains high current tracking accuracy. After PI-LADRC is used, the THD of incoming current decreases from 3.28% to 2.47%. In contrast, traditional PI-PR control results in a high proportion of higher-order harmonics in the output current, which may adversely affect the grid-connected system.

**Table 2:** THD value comparison table.

controller	THD(100%)	
	15A	30A
PI-PR	3.28	2.47
PI-LADRC	1.63	1.22

**Fig 9:**
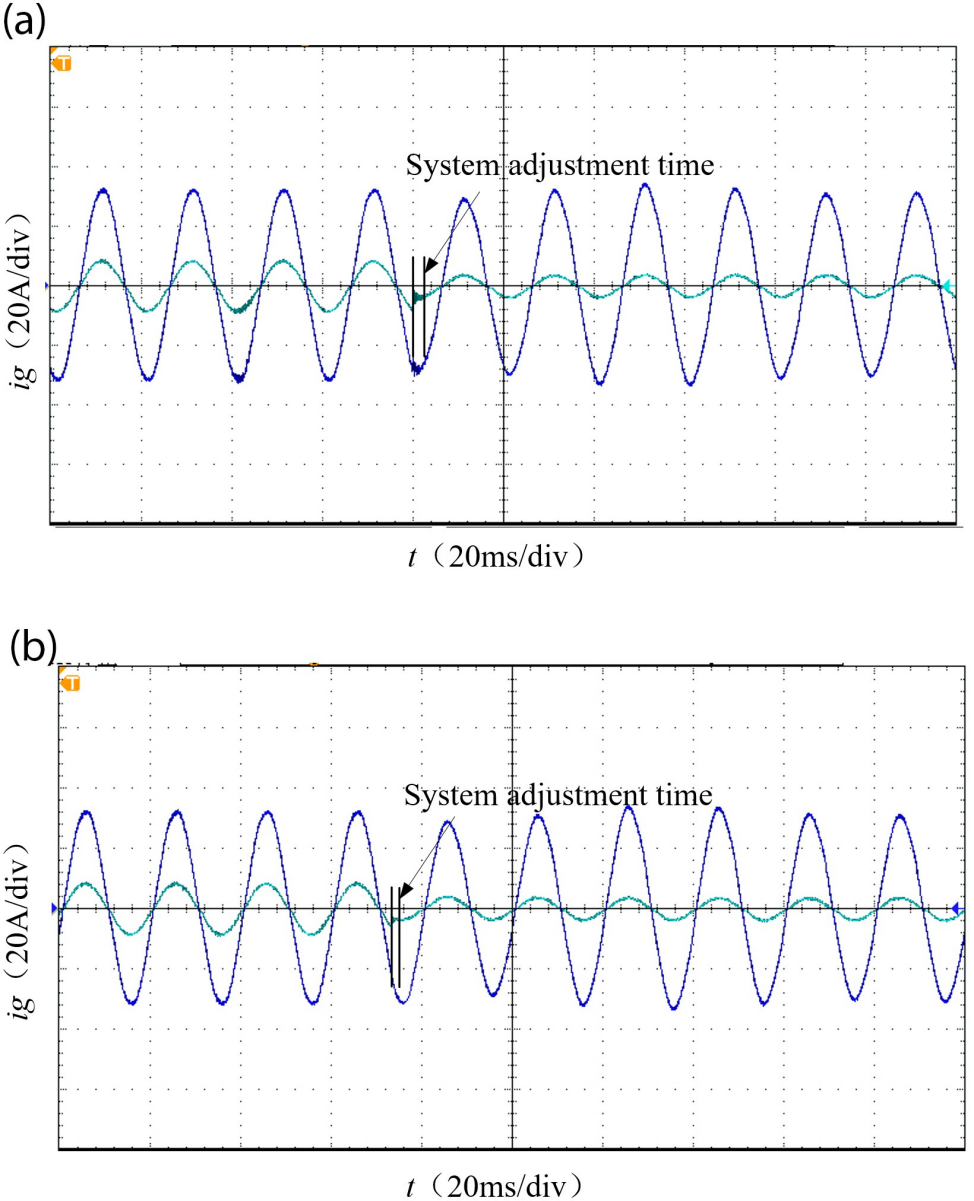
Current step response waveform. (a) PI-PR. (b) PI-LADRC.

To verify that the adopted PI-LADRC method performs better in terms of dynamic performance compared to PI-PR, we operate the grid-connected inverter under the condition that only the control method differs while keeping all other parameters the same. When the set value of the grid current changes abruptly from 9A to 4A, the waveform of the grid current output is shown in Fig 9.

Based on Fig 9, it is evident that the PI-LADRC control exhibits a significant reduction in system settling time compared to the PI-PR control when subjected to sudden variations in current. Specifically, the PI-LADRC control achieves a two-thirds reduction in settling time. This observation highlights the superior dynamic performance of PI-LADRC control over PI-PR control while effectively preserving its excellent disturbance rejection capabilities.

To demonstrate the effectiveness of the proposed PI-LADRC method, we have shown through simulation and experimental validation that it effectively mitigates issues such as poor grid current quality, higher-order harmonics, and insufficient anti-interference capability, which are commonly associated with traditional PI-PR control. The results indicate that the PI-LADRC control not only offers improved disturbance rejection capability and control accuracy but also exhibits superior dynamic performance, with a significant reduction in system settling time compared to traditional PI-PR control. These findings underscore the potential of the PI-LADRC method in addressing critical challenges in grid-connected inverter control.

## Conclusion

This paper presents a novel control approach, specifically a grid-connected control strategy for *LCL*-type single-phase inverters based on a reduced-order LADRC. The incorporation of a proportional-integral controller at the front end significantly enhances the system’s capability to suppress grid harmonics and reduce the total harmonic distortion of the current. Results from experiments and simulations demonstrate that the proposed current controller can ensure rapid transient response and excellent steady-state performance in the presence of parameter uncertainty and disturbances.

Moreover, future research for this study will focus on investigating the practical implementation of real-time optimization techniques to enhance the performance and efficiency of the control strategy in grid-connected photovoltaic systems.

## Supporting information

S1 Fig PC-based supervisory control. (TIF)

S2 Fig PI-LADRC control simulation model. (TIF)

S3 Fig PI-PR control simulation model. (TIF)
