# Supplementary figures and images for: LADRC-based grid-connected control strategy for single-phase LCL-type inverters
Source: PLoS One. 2024 May 15;19(5):e0303591. doi: 10.1371/journal.pone.0303591 (PMC11095700; doi:10.1371/journal.pone.0303591)

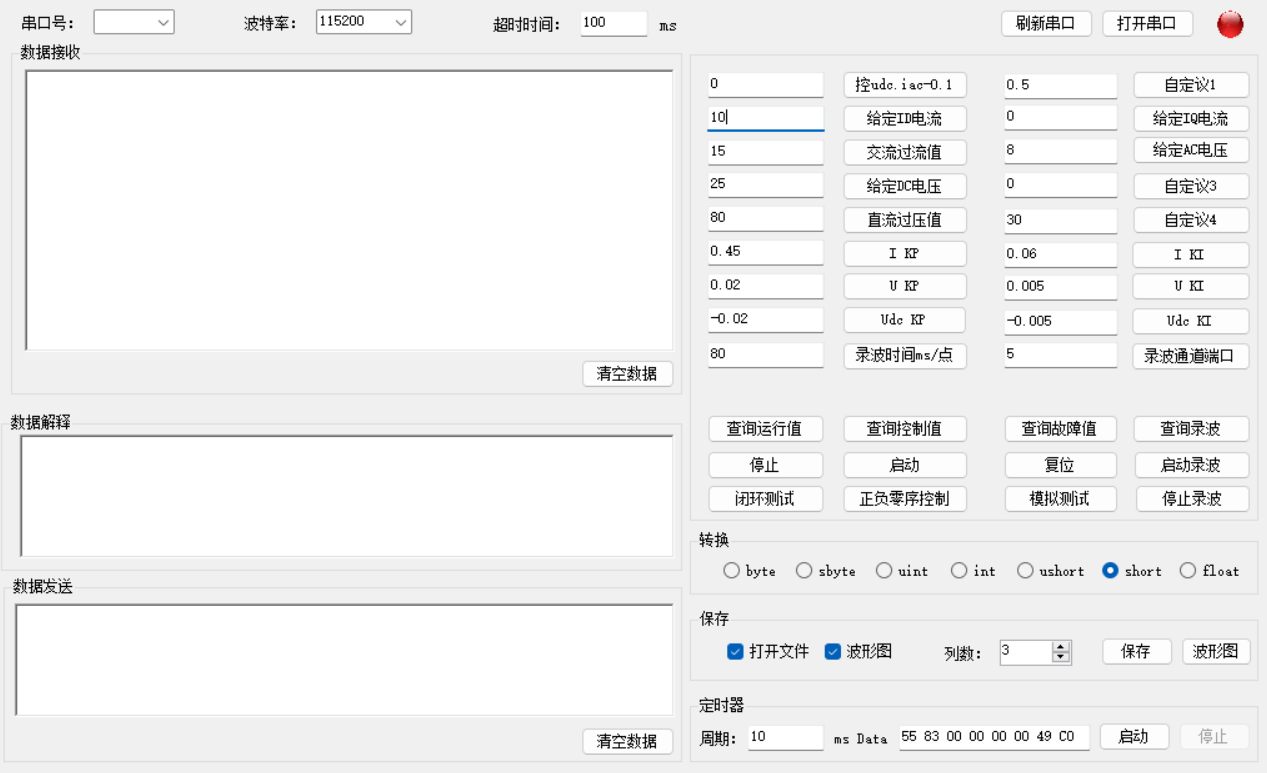

Supplement: S1 Fig — (TIF) [file pone.0303591.s001.tif]

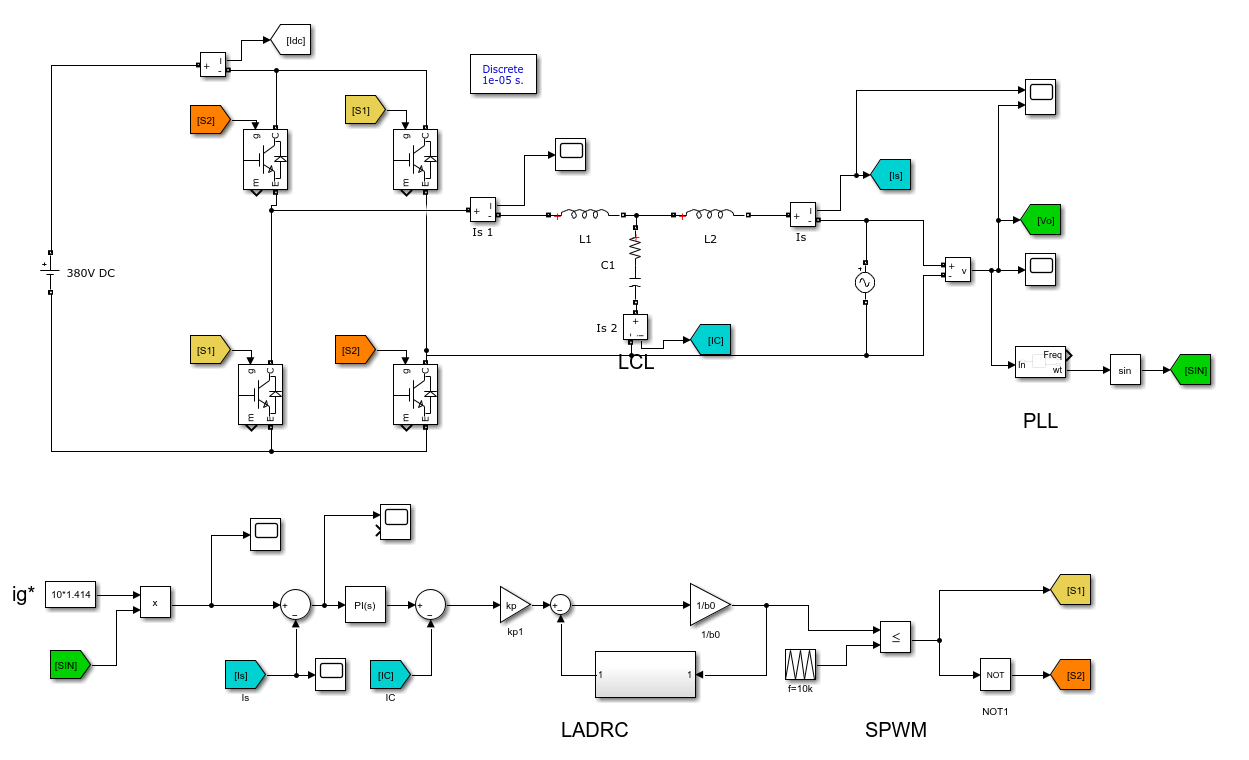

Supplement: S2 Fig — (TIF) [file pone.0303591.s002.tif]

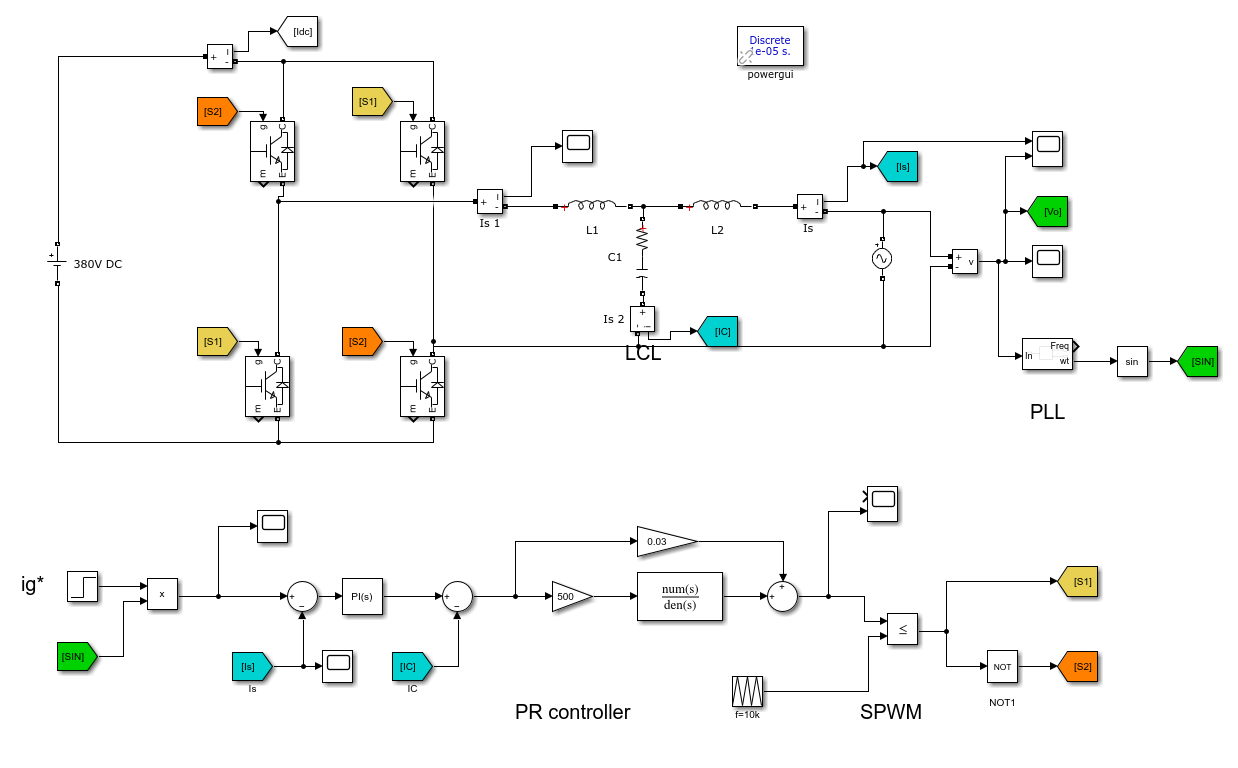

Supplement: S3 Fig — (TIF) [file pone.0303591.s003.tif]
